# Frequency of employer changes and their financial return: gender differences amongst German university graduates

**DOI:** 10.1186/s12651-017-0235-3

**Published:** 2018-01-02

**Authors:** Johannes Wieschke

**Affiliations:** Bayerisches Staatsinstitut für Hochschulforschung und Hochschulplanung (IHF), Prinzregentenstraße 24, 80538 Munich, Germany

**Keywords:** Employer change, Occupational mobility, Gender wage gap, University graduates, Income development, J31

## Abstract

Gender differences in the frequency of employer changes and their financial return were examined in a sample of Bavarian university graduates. The search and matching theories were used to develop hypotheses which were then tested against each other. The results show that in the first few years after graduation women change employer more frequently than men. In large part this can be explained by gender differences in labor market structures, in particular the fact that a woman’s first job is less likely to be in a large company, in an executive position or on a permanent contract and women tend to be less satisfied with their first job. After controlling for variance in these factors the coefficient changes sign, indicating that under similar circumstances men change employer more often. Furthermore, both men and women benefit financially from changing employer. The absolute return is higher for men, but as men tend to have a higher starting salary there is no gender difference in the relative return and hence no effect on the gender gap. The results are also discussed in the light of the specifics of the structure of the German labor market.

## Zusammenfassung

Diese Studie nutzt eine Stichprobe bayerischer Hochschulabsolventen, um Geschlechtsunterschiede sowohl in der Häufigkeit als auch in den finanziellen Erträgen von Arbeitgeberwechseln zu untersuchen. Die Such- und Matchingtheorien werden genutzt, um Hypothesen zu entwickeln, die anschließend gegeneinander getestet werden. Die Ergebnisse zeigen, dass Frauen ihren Arbeitgeber in den ersten Jahren nach dem Abschluss häufiger wechseln als Männer. Das kann zu einem großen Teil durch unterschiedliche Arbeitsmarktstrukturen erklärt werden, vor allem durch die Tatsache, dass Frauen in ihrer ersten Beschäftigung seltener in Großbetrieben, in Führungspositionen und mit unbefristeten Verträgen arbeiten und weniger zufrieden mit ihrer Arbeit sind. Sobald auf all diese Faktoren kontrolliert wird, ändert der Koeffizient sein Vorzeichen, d. h. unter gleichen Umständen wechseln Männer den Arbeitgeber häufiger. Weiterhin profitieren sowohl Männer als auch Frauen finanziell von Arbeitgeberwechseln. Männer erhalten höhere absolute Erträge, aber aufgrund ihrer höheren Einstiegseinkommen existiert bei den relativen Erträgen kein Unterschied zwischen Männern und Frauen und somit auch kein Einfluss auf die Gender Pay Gap. Die Ergebnisse werden vor dem Hintergrund der Besonderheiten des deutschen Arbeitsmarktes diskutiert.

## Introduction

Differences between men and women are widely discussed with regard to the labor market. A significant part of the sociological and economic literature concentrates on explaining the gender differences in wages, sometimes referred to as the gender pay gap (GPG). These differences vary over countries, cohorts and time spans, but are found almost everywhere and almost all the time (Gartner and Hinz 2009: 566; Mandel and Semyonov 2005: 957; Weinberger and Kuhn 2010: 389; Triventi 2013: 571; Kassenboehmer and Sinning 2014: 339). There are several theories and models which account for a substantial part of the gender pay gap, e.g. by including differences in human capital endowments.

One aspect of the gender pay gap that has been neglected thus far in the German context are the potential gender-specificities in job mobility, the characteristics and effects of which have been explored in several previous studies. However, most of these studies were based on data from Anglophone countries with flexible labor markets and they have produced mixed results. For example, there is evidence that moving directly from one job to another has a beneficial effect on incomes (Keith and McWilliams 1999), but another study suggested that indirect job transition also has positive effects (Antel 1991). One factor that probably influences these mechanisms is the labor market structure as described in the varieties of capitalism literature. One would therefore expect analyses of German data to yield different results since Germany is usually classed as having a coordinated market economy (Hall and Soskice 2001: 21 f.) and there is currently less evidence on the mechanisms underpinning the gender pay gap in such economies. The main features of the German labor market include a high segmentation on the basis of qualifications and skills, low mobility between segments (Scherer 2004: 373) and high employment protection (Hall and Soskice 2001: 19). These factors probably affect the frequency of job changes and their outcomes which makes it important to analyze coordinated market economies as well.

Given the rising number of university graduates and the importance of the early years of an individual’s employment—when wage growth is especially strong (Fuller 2008: 158) but the influence of family not yet very pronounced (Triventi et al. 2015: 26)—the population analyzed in this study has particular importance.

The issues on which this study focused were the frequency with which individuals change employer during their early career, the nature of the relationship between gender and changes of employer and how changes of employer affect wages and the gender pay gap in Germany. Thus the results can be compared with those of other studies to provide an analysis of the effects of labor market structure.

## Theory and state of research

The search and matching theories provide the theoretical foundation for this study; they apply not only to the search for a first job, but to subsequent job changes as well. According to these theories, individuals try to find a job that matches their preferences and abilities as closely as possible in order to maximize the financial and non-financial returns of work. Employers, too, are looking for the optimal match between post and employee for the same reasons (Scherer 2005: 428). However, potential employees have only limited information about the labor market (Jovanovic 1979: 973) so every job search involves investing money, time and other resources, hence job searches have costs (Wilde 1981: 1124). These costs rise with the effort made but are also positively correlated with the number of posts considered. According to the theory, individuals will search as long as the expected returns of the search exceed the costs.

Because search costs are not exclusively financial and because income is only one of several important characteristics of a job one would expect different people in the same situation to use different searching behaviors and this makes searching behavior hard to predict. Gender differences in labor market preferences (Daymont and Andrisani 1984: 414) may thus also contribute to differences in job mobility (Ng et al. 2007). Generally speaking, however, the probability of an individual changing job should be negatively correlated with the quality of their current job, because the higher the quality of one’s current job, the fewer the number of better jobs. The following analyses focus on objective job characteristics because data on individual preferences are not available.

Due to educational and, following this, occupational selectivity, men are more likely than women to work in sectors in which there is a strong relationship between education and occupation (e.g. engineering), so it is easier for them to find a good match. Furthermore, because women tend to have lower incomes even at the beginning of their occupational career (Kunze 2005: 87; Leuze and Strauß 2014: 286) it should, other things being equal, be easier for women in their first job to find a better one. However, this assumption possibly cannot (or only to a limited extent) be confirmed when occupational segregation, which can also lead to lower incomes for women, is considered: Because it is often only possible to move to another occupational sector if one acquires the appropriate qualifications, not every job is available to everyone without an interruption in employment (Schiener 2006: 133 f.). This is especially important in the German context since the German labor market is characterized by stronger segmentation than, for example the British one. In Germany, academic degrees and “occupationally defined fields” play an important role in separating sectors of the labor market from one another. This also makes “entrapment scenarios” (Scherer 2004: 373 f.) in which suboptimal entry jobs have long-lasting negative effects on career (Scherer 2004: 378) more likely.

Thus it is rather likely that due to self-selection, individuals with unsatisfactory jobs are more likely than their peers with satisfactory jobs to be employed in sectors with less attractive workplaces, i.e. gender differences in working conditions can be (partly) explained by the gender distribution of employees across the various sectors. This could be an important explaining factor when gender differences in employer change frequencies cannot be found. In fact there is evidence that occupational segregation by gender is decreasing, but gender differences in the labor market and in choice of academic subjects are still present (Charles and Bradley 2009: 941; Blau et al. 2013: 481).

Furthermore, there are various starting points from which the structure of the labor market can lead to gender-specific effects of employer change on income. One possibility, for example, is that there are better career prospects in occupational sectors where there is more vertical differentiation between jobs. If men and women are unevenly distributed over such labor markets then changing employer could, on average, yield different results for men and women.

Another possibility, however, is the predominance of the effect of the entry job. If the incomes are low in this job, it is easier to increase one’s income by changing job than in a comparison group where incomes are already higher before the change. On the other hand, people who accept a lower starting salary may have a lower target income. These assumptions lead to different hypotheses, which are then tested against each other in the empirical section.

Previous studies did not find a gender difference in the frequency of employer change; however they did find a gender difference in the returns of employer changes. Men seem to benefit to more from changing employer than women (Loprest 1992; Del Bono and Vuri 2011; Johnston and Lee 2012; Merluzzi and Dobrev 2015). Both the frequency of job change and its return should, therefore, be examined in an analysis of the temporal changes in the gender pay gap.

## Hypotheses

**Fig. 1:**
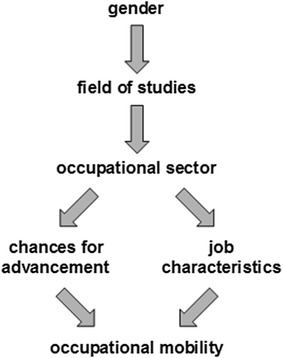
Assumed relationships (*Source* Author; created with LibreOffice Draw 4.3)

The arguments outlined in Sect. 2 imply that two factors should be considered in an analysis of possible gender differences: the first step is to ask whether men and women differ with respect to the frequency of employer changes and what factors are responsible for any such difference. Figure 1 is a directed acyclic graph showing the assumed causal effects. There are gender differences in the distribution of employees across occupational sectors (e.g. 18% of women and 42% of men find their first job in the manufacturing sector)—amongst other reasons this is due to gender differences in choices of field of study. There are also sector differences in chances of advancement as a result of changing employer. Similarly, on average different starting positions with regard to certain job characteristics—e.g. the frequency of permanent contracts or the firm size have been shown to influence not only income (Orlowski and Riphahn 2011: 38) but also the probability of employer change (Dütsch and Struck 2014: 116). Employer changes are thus affected by two factors—chance of advancement and job characteristics—although it is assumed that these work in different directions. The following analyses were intended to reveal which factor is the more important.

Two hypotheses were therefore tested against each other:

1. Women change their employer more often than men because, for the same search cost, their on average worse starting position means that it is more likely they will benefit from doing so, e.g. in the form of a higher salary or a better match between the job and their qualifications (*H1* search gain hypothesis).
2. Women do not change their employer more often than men because segregation of the labor market means that it is not easier for them to find a better job for the same search cost, despite their on average worse starting position (*H2* segregation hypothesis).

It should also be noted that the question whether women change their employer more often than men is rather descriptive because even hypothesis 1 does not state that women are inherently more mobile. The hypotheses instead concentrate on the reasons for potentially higher job mobility among women.

The second step of the analysis deals with the financial consequences of employer change rather than the frequency of such changes. The question addressed here is whether there are gender differences in the return on employer changes. Again, there are two conflicting hypotheses:

1. Women benefit more from changing employer than men because their generally worse starting position makes it easier for them to achieve a wage increase in this way (*H3* entry job hypothesis).
2. Men benefit more from changing job than women because they are more likely to be employed in a sector where the chances of advancement through job mobility are good (*H4* advancement hypothesis).

In both cases the effects probably cannot be attributed to one single factor (e.g. the entry job or promotion opportunities); it is likely that several factors are at work simultaneously, possibly acting in different directions.

## Data

The data used in the analysis were obtained from the Bavarian Graduate Panel (BAP—Bayerisches Absolventenpanel). This panel consists of cohorts of university graduates who are recruited about every 4 years and then questioned several times, at about 1, 5 and 10 years after graduation. The statistical population consists of all graduates of the universities and public universities of applied sciences in Bavaria in the selected year. A comprehensive survey is always conducted in order to gain a sample of Bavarian graduates which is as representative as possible. Previous research has shown that there are only minor differences between the data from the BAP and the DZHW^1^ graduate panel which is recruited from the population of all German graduates (Falk et al. 2014: 8 ff.).

The following analysis is based on the 2005/06 graduate cohort. To date this cohort has been surveyed twice, so information about their academic studies and the first years of their occupational career is available. Occupational data are recorded to within a month, so it is possible to reconstruct income dynamics and assign them to different jobs. Individuals may change job whilst remaining with the same employer (e.g. indicated by a change in income or working hours), but since the objects of investigation in this analysis were the frequency and effects of employer changes, the term “job mobility” is used only to refer to changes of employer, not to job changes within a company.^2^

Although the data cannot be generalized to the entire population since only people with tertiary education were questioned, they have several advantages over other data sets. As shown in previous studies, the gender gap in wage growth—and wage growth itself—is a phenomenon that is particularly pronounced amongst university graduates (Johnston and Lee 2012: 135 f.). This analysis of graduates should, therefore, contribute substantially to understanding of the gender pay gap.

The time span analyzed here—the years immediately after graduation—is also of particular interest, since a high proportion of income growth across the career is achieved in the early career (Fuller 2008: 158) and because interruptions in employment for family reasons are not very common in this period. Furthermore, important variables are available to a high level of precision: income is described as a metric variable and information about employment characteristics is given on a monthly basis, from the date of graduation. Thus both the emergence and the development of the gender pay gap amongst university graduates can be tracked very precisely.

Initially, the sample consisted of 3325 individuals with 222,446 person months (66.9 observations per person, on average). Observations with missing values, episodes with a gross monthly income of less than 400 euros, episodes where gross hourly pay exceeded 100 euros and episodes of self-employment (for which only net income data are available) were dropped from the sample. When this had been done the dataset consisted of 2258 persons (1001 women and 1257 men) and 146,817 observations (65 per person).

## Descriptive statistics

**Fig. 2:**
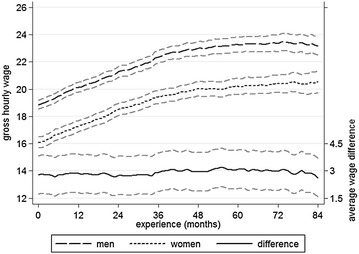
Changes in pay by gender: gross hourly wages with 95% CIs (*Source* BAP 2005/06, author’s calculations; performed with Stata13)

This section presents a descriptive overview over the gender pay gap in the sample. Figure 2 shows the trends in gross hourly wages for men and women as their experience increases, beginning with the first job after graduation. Hourly wages are used instead of monthly income to control for differences in working hours. On average women do less paid work than men (Kleiner et al. 2015: 103; in this sample, the gender differences in average contractual and actual working hours per week amounted to about 1.5–3.5 and 2.5–5.5 h, respectively; gender differences in working hours also tended to increase with work experience), so the relative difference in hourly wages is lower than that for monthly income. Immediately after graduation the difference in hourly wages amounts to approximately three euros. It slightly changes over the following years, but never differs much from this starting value. A large part of the gender difference in income is thus already present at the beginning of the working career. Over the observation period absolute wages rose from 18.9 to 23.2 euros for men (+ 22.9%) and from 16.1 to 20.6 euros for women (+ 27.8%).

Table 1 contains several statistics that show the development of absolute and relative incomes. In order to control for outliers, not the average incomes with work experiences of 0 and 84 months are used, but the averages over months 0–11 and 73–84. As can be seen, in absolute terms the pay gap widens, both when examining monthly income and hourly wages. The difference in monthly income rises from 19.6 to 20.9%, whereas the difference in hourly wages drops from 14.4 to 12.5%. Subsequent analyses of the changes in income and the gender pay gap take this into account in order to provide as complete a picture as possible. It should also be noted that the incomes reported here are probably not representative of Germany as a whole, since average wages in Bavaria exceed those in other parts of Germany (Eichhorn et al. 2010: 291).

**Table 1:** Changes in income. *Source* BAP 2005/06, author’s calculations

	Months 0–11	Months 73–84	Abs. change	Rel. change (%)
Monthly income				
Men	3261 €	4032 €	771 €	23.7
Women	2621 €	3189 €	568 €	21.7
GPG (€)	640 €	843 €	203 €	31.9
GPG (%)	19.6%	20.9%	1.3 pp	6.7
Hourly wages				
Men	19.44 €	23.36 €	3.92 €	20.2
Women	16.64 €	20.45 €	3.81 €	22.9
GPG (€)	2.80 €	2.91 €	0.11 €	3.9
GPG (%)	14.4%	12.5%	− 1.9 pp	− 13.5

*GPG* gender pay gap, *pp* percentage points

Further descriptive statistics can be found in Tables 2 and 3, which give average values for the time-constant (2) and time-varying 3) independent variables, separated by gender, employer change and—in case of the time varying variables in Table 3—work experience. This makes it possible to identify differences and trace important changes over time. The first set of variables includes university (vs. university of applied sciences) and field of study (five categories) both of which are important predictors of subsequent position in the labor market. The variable parental academic background (i.e. at least one parent vs. no parents with a university degree) is used to capture respondents’ social origin. Study abroad, which captures previous mobility experiences, is of importance mainly for the regressions on employer changes. Number of semesters and final grade (which for multivariate analysis is standardized over field of study and inverted so that higher values indicate better grades) are indicators of academic performance and are expected to influence wages.

**Table 2:** Time-constant sample characteristics by gender and employer change. *Source* BAP 2005/06, author’s calculations

	Female	Male
University		
No change	0.57 (0.50)	0.43 (0.50)
Change	0.63 (0.48)	0.53 (0.50)
Field of study		
Language/cultural		
No change	0.21 (0.41)	0.04 (0.19)
Change	0.25 (0.44)	0.08 (0.27)
Social sciences		
No change	0.18 (0.38)	0.03 (0.18)
Change	0.16 (0.37)	0.03 (0.17)
Law/economics		
No change	0.33 (0.47)	0.33 (0.47)
Change	0.37 (0.48)	0.35 (0.48)
Math/sciences		
No change	0.18 (0.38)	0.22 (0.42)
Change	0.16 (0.37)	0.25 (0.43)
Engineering		
No change	0.10 (0.30)	0.38 (0.48)
Change	0.05 (0.22)	0.30 (0.46)
Academic background		
No change	0.50 (0.50)	0.43 (0.50)
Change	0.54 (0.50)	0.47 (0.50)
Study abroad		
No change	0.38 (0.49)	0.35 (0.48)
Change	0.45 (0.50)	0.44 (0.50)
Semesters		
No change	9.83 (1.63)	9.72 (1.73)
Change	9.87 (1.65)	9.88 (1.73)
Final grade		
No change	1.84 (0.49)	1.92 (0.50)
Change	1.82 (0.48)	1.89 (0.50)
Satisfaction 1st job^a^		
No change	3.91 (1.03)	4.10 (0.88)
Change	3.50 (1.07)	3.74 (1.05)
*N*		
No change	467	645
Change	455	544

Proportions and means with standard deviations in parentheses

^a^Satisfaction: five-point scale with 1 = lowest and 5 = highest

**Table 3:** Time-variant sample characteristics by gender, employer change and work experience. *Source* BAP 2005/06, author’s calculations

	Exp = minimum		Exp = maximum	
	Female	Male	Female	Male
Occupational sector				
1: BIC				
No change	0.12 (0.33)	0.12 (0.32)	0.12 (0.33)	0.12 (0.32)
Change	0.10 (0.31)	0.14 (0.35)	0.07 (0.26)	0.13 (0.33)
2: Manufacturing				
No change	0.21 (0.41)	0.48 (0.50)	0.21 (0.41)	0.49 (0.50)
Change	0.16 (0.36)	0.34 (0.47)	0.21 (0.41)	0.44 (0.50)
3: Services				
No change	0.42 (0.49)	0.26 (0.44)	0.41 (0.49)	0.26 (0.44)
Change	0.44 (0.50)	0.28 (0.45)	0.43 (0.49)	0.26 (0.44)
4: Media et al.^a^				
No change	0.25 (0.43)	0.14 (0.34)	0.25 (0.44)	0.14 (0.34)
Change	0.30 (0.46)	0.23 (0.42)	0.29 (0.46)	0.17 (0.38)
Firm size (employees)				
Small (< 100)				
No change	0.36 (0.48)	0.21 (0.41)	0.36 (0.48)	0.22 (0.41)
Change	0.42 (0.49)	0.31 (0.46)	0.34 (0.47)	0.22 (0.42)
Medium (100–499)				
No change	0.17 (0.38)	0.14 (0.34)	0.17 (0.37)	0.14 (0.34)
Change	0.21 (0.41)	0.20 (0.40)	0.22 (0.41)	0.16 (0.37)
Large (≥ 500)				
No change	0.47 (0.50)	0.65 (0.48)	0.47 (0.50)	0.65 (0.48)
Change	0.37 (0.48)	0.49 (0.50)	0.45 (0.50)	0.62 (0.49)
Executive position				
No change	0.24 (0.43)	0.35 (0.48)	0.33 (0.47)	0.44 (0.50)
Change	0.12 (0.33)	0.18 (0.39)	0.30 (0.46)	0.45 (0.50)
Public sector				
No change	0.42 (0.49)	0.29 (0.45)	0.43 (0.50)	0.29 (0.45)
Change	0.38 (0.49)	0.32 (0.47)	0.45 (0.50)	0.33 (0.47)
Permanent contract				
No change	0.78 (0.41)	0.85 (0.35)	0.82 (0.39)	0.87 (0.34)
Change	0.53 (0.50)	0.67 (0.47)	0.78 (0.41)	0.86 (0.34)
Multinat. company				
No change	0.49 (0.50)	0.67 (0.47)	0.49 (0.50)	0.66 (0.47)
Change	0.39 (0.49)	0.54 (0.50)	0.43 (0.50)	0.63 (0.48)
Part-time (< 30 h/week)				
No change	0.14 (0.34)	0.03 (0.18)	0.15 (0.36)	0.02 (0.15)
Change	0.14 (0.35)	0.09 (0.28)	0.16 (0.36)	0.03 (0.17)
Wage (€/h)				
No change	17.31 (6.77)	19.77 (5.51)	18.61 (7.35)	21.5 (7.59)
Change	15.37 (5.92)	18.43 (6.43)	22.09 (7.95)	27.6 (9.39)
*N*				
No change	473	650	473	650
Change	528	607	528	607

Shares and means with standard deviations in parentheses

*BIC* banks, insurances, consulting

^a^Media et al.: Media, education, associations

Table 3 gives data on occupational sector (four categories), firm size and dummy variables for holding an executive position, public sector employment, employment on a permanent contract, employment with a multinational company and part-time employment. These job characteristics have a major impact on income and should also influence individuals’ willingness or need to change employer. Gross hourly wages and the mean values for overall job satisfaction in one’s first job (1 = lowest satisfaction and 5 = highest) are included.

Table 2 thus shows, for example, that people who changed employer are more likely to have studied abroad and at universities; people who do not change employer are more likely to have attended universities of applied sciences. These facts seem to highlight the importance of previous mobility experiences for future mobility (David et al. 2010: 201): For students at universities—compared to those at universities of applied sciences—there are on average greater distances between the places of their secondary and tertiary education (Kratz and Lenz 2015: 13). Hence university students may more often make mobility experiences which, through learning-by-doing effects, could enhance future (job) mobility (DaVanzo 1981: 46). Previous research has also shown that internationally mobile students tend change employer more often than those who did not study abroad (Kratz and Netz 2016: 17).

Even more interesting are the statistics presented in Table 3, which gives respondents’ characteristics at their first and last observation. As can be seen from columns 1 and 2, individuals who do not change employer during the observation period initially have a wage advantage of about 1–2 euros per hour, but as work experience increases this becomes a disadvantage of several euros per hour; the disadvantage is especially pronounced for men. Other variables also show major shifts. The proportion of mobile men working in small companies falls from 31 to 22%, whilst the proportion working in large companies rises from 49 to 62%, a change that probably contributes to the income variations described above.

The chance of being on a permanent contract increases for both men and women—especially if they change job. There are also gender-specific developments in working hours. About 14–16% of both mobile and immobile women work part-time (less than 30 h per week), both at the beginning of their career and after several years. However, the proportion of men in part-time work falls, from 9 to 3% for those who change job and from 3 to 2% for those who do not.

## Analysis

### Frequency of employer change

The first step of the analysis presented here investigates the relationship between gender and the frequency of employer changes. One hypothesis was that women would be more likely to change job, because for a given search cost they are more likely to gain an advantage from changing employer (*H1* search gain hypothesis). The competing hypothesis assumes that this is not the case due to gender differences in job availability and segregation in the labor market (*H2* segregation hypothesis).

Table 4 gives the frequencies (by gender and overall) for total number of employer changes during the observation period. As can be seen from the third column, about half the sample did not change employer over this period and about a third changed employer just once. Less than one-fifth of the sample changed employer more than once and less than 1% reported the maximum of four changes.

**Table 4:** Final number of employers by gender. *Source* BAP 2005/06, author’s calculations

Total number of employer changes	Female (%)	Male (%)	Total (%)	Cumulative (%)
0	47.25	51.71	49.73	49.73
1	30.67	32.94	31.93	81.67
2	14.89	12.41	13.51	95.17
3	6.19	2.47	4.12	99.29
4	1.00	0.48	0.71	100.00
Total	100.00	100.00	100.00	

*N* = 2258; χ^2^: *p* = 0.000; Cramér’s V: 0.109

There were some gender differences in employer mobility. Almost 52% of men did not change employer during this period and 33% did so only once, whereas the corresponding figures for women are about 47 and 31%, a cumulative difference of about 7% points, Women are over-represented in all the remaining employer mobility categories (although on average women reported only 63.5 working months whereas men reported 67.9), yielding averages of 1.83 employers for women and 1.67 for men. A Chi squared test yielded a highly significant result, *p* = 0.000, but Cramér’s V = 0.109 indicates only a weak relationship between gender and the number of employer changes.

Next cross-sectional logistic regressions were used for a multivariate analysis of the effect of gender on the probability of changing employer at least once during the observation period. The values of the first observations, when the individuals had just entered the labor market, were used in this analysis. In this methodological context, however, nested models can be problematic because their β-coefficients refer to differently scaled dependent variables and cannot, therefore, be compared with each other (Best and Wolf 2012: 383; Mood 2010: 72). For this reason, average marginal effects are reported, as these can be compared across different nested models (Best and Wolf 2012: 388; Mood 2010: 80). Table 5 contains the results of the estimated regression models.

**Table 5:** Average marginal effects for logistic regressions on employer change. *Source* BAP 2005/06, author’s calculations

Employer change	Model 1	Model 2	Model 3	Model 4
Male	− 0.045*	− 0.002	− 0.008	0.044*
University		0.040	0.016	0.016
Field of study (FoS)				
Language/cultural		(Reference)	(Reference)	(Reference)
Social sciences		− 0.055	− 0.070	− 0.068
Law/economics		− 0.073*	− 0.044	− 0.007
Math/sciences		− 0.073	− 0.076*	− 0.047
Engineering		− 0.143**	− 0.098*	− 0.053
Acad. background		0.017	0.018	0.019
Study abroad		0.072**	0.071**	0.069**
Semester		0.001	0.000	− 0.009
Over FoS: std. grade		− 0.005	− 0.009	− 0.003
Occupational sector				
1: BIC			0.056	0.067
2: Manufacturing			(Reference)	(Reference)
3: Services			0.088**	0.031
4: Media et al.^a^			0.147***	0.010
Firm size (employees)				
Small (< 100)				− 0.015
Medium (100–499)				(Reference)
Large (≥ 500)				− 0.108***
Executive position				− 0.156***
Public sector				− 0.077**
Permanent contract				− 0.241***
Multinat. company				− 0.016
Part-time				0.005
Hourly wage				− 0.002
Job satisfaction				
1: Very low				(Reference)
2				0.058
3				− 0.022
4				− 0.109*
5: Very high				− 0.196***
Constant	0.503***	0.503***	0.503***	0.503***

*BIC* banks, insurances, consulting

*N*: 2258; * p ≤ 0.05, ** p ≤ 0.01, *** p ≤ 0.001

^a^Media et al.: Media, education, associations

In the raw model without any control variables the gender coefficient is negative and significant, indicating that men were somewhat less likely than women to change employer at least once. Models 2–4 include several control variables: personal and study characteristics in model 2, occupational sector in model 3 and further job characteristics in model 4. As can be seen from the relevant columns, the inclusion of personal and study characteristics reduces the value of the gender coefficient to insignificance. This change is observed even when only the university or the field of study variable is included. Since the relationship between education and occupation is particularly strong in the case of engineering, it is not surprising that the largest negative effect was found for this field of study. Adding occupational sector as a control variable does not have a strong effect on the gender coefficient, but it does reduce the importance of the field of study, as one would expect given the connections shown in Fig. 1. Both field of study and occupational sector thus act as intervening variables.

Finally, both field of study and occupational sector do not have significant coefficients once the other job characteristics are accounted for. This indicates that these job characteristics are more important predictors of employer changes than the remaining aspects of occupational sector, namely career prospects. In the full model gender has a significant coefficient, *p* = 0.044, but the sign has changed, indicating that given the same personal and occupational background, men were more likely to leave their first employer than women. The greatest effects—all of which make employer changes less likely—were associated with having a permanent contract, holding an executive position, working for a large company and overall job satisfaction. Only once all four of these variables were included did the gender coefficient become positive and significant. Other variables that exerted a significant influence were studying abroad, which made employer change more likely, and employment in the public sector, which made it less likely. As women were over-represented in the public sector including this variable reduces the gender coefficient and increases the *p* value, but not above the threshold of 0.05.

**Fig. 3:**
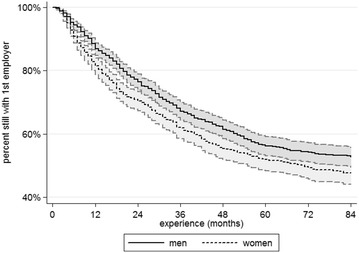
Gender-specific job mobility: time course of first employer change and 95% CIs (*Source* BAP 2005/06, author’s calculations; created with Stata13)

**Fig. 4:**
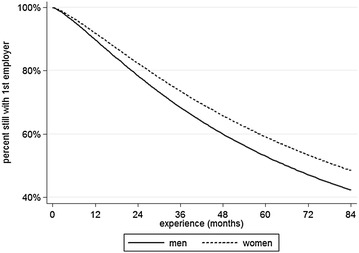
Gender-specific job mobility: time course of first employer change (*Source* BAP 2005/06, author’s calculations, control variables included; created with Stata13)

Additionally, an event history analysis was conducted to provide a more detailed picture of the changes. Kaplan–Meier survival curves were calculated for the proportions of male and female respondents who still worked for their first employer. As can be seen in Fig. 3, the curve for men is slightly above that for women—especially in the first 2 years on the labor market—indicating that men are slower to leave their first employer. A test of group differences yields a significant p-value of 0.008. This difference is reversed as soon as the effects of other independent variables are controlled for in a log-logistic regression; the gender coefficient is significant here, and the relationship between gender and employer change is shown in Fig. 4. Once the independent variables are included the women’s curve sits above the men’s curve, indicating that men leave their first employer more quickly if control variables are included.^3^

The first of the two competing hypotheses predicts higher female job mobility due to their worse starting position (*H1* search gain hypothesis) and seems to be supported by the results. In the cross-sectional logistic regressions, the negative gender coefficient in model 1 is highly significant and changes algebraic sign only once personal, study and employment characteristics are included. This indicates that women are not inherently more mobile than men; their greater mobility is a reflection of certain occupational sector-related gender differences. Women are less likely to start their working career with a permanent contract, in an executive position or in a large company and these variables play large and significant roles in employer mobility. At the same time neither the coefficient for field of study nor those for most of the occupational sectors were statistically significant. The large coefficient for permanent contracts, suggests that this—alongside the gains to be made from changing employer—is another important determinant of job mobility. If an employer does not offer an extension to a fixed or temporary contract or an alternative post within the company one has to change employer in order to avoid unemployment. When men and women with the same characteristics are compared, however, men seem to be more mobile; the explanation for this may lie in gendered labor market preferences. Men and women differ in how they value job characteristics such as remuneration and working hours (Daymont and Andrisani 1984: 414; Fortin 2005: 425), which may make men more likely to leave a job than women, if other factors are equal; this would be reflected in a significant gender coefficient. Were it possible to control for individual differences in labor market preferences and not just for objective job characteristics this gender difference in job mobility might disappear, since previous research has shown that several subjective criteria play an important role in explaining differences in turnover intentions (Sousa-Poza and Henneberger 2004: 131).

Further analyses are necessary to provide a more complete picture of the effects of male and female job mobility, so the next step in the analysis was an investigation of gender-specific returns on employer changes.

### Return on employer change

This section begins with a descriptive examination of the relevant data. One hypothesis is that women benefit more from job mobility than men because their on average worse starting positions make it easier for them to increase their pay (*H3* entry job hypothesis), the other hypothesis is that men benefit more, because they are more likely to be employed in sectors with good prospects for advancement (*H4* advancement hypothesis).

**Fig. 5:**
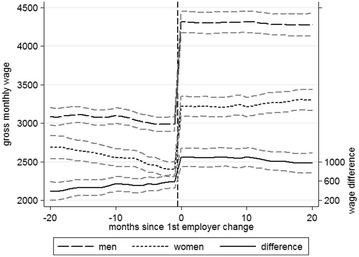
Gender-specific changes in income: monthly income of employer changers and 95% CIs (*Source* BAP 2005/06, author’s calculations; created with Stata13)

**Fig. 6:**
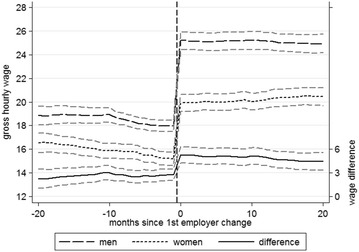
Gender-specific changes in pay: hourly wages of employer changers and 95% CIs (*Source* BAP 2005/06, author’s calculations; created with Stata13)

Figures 5 and 6 show the average monthly incomes and hourly wages for men and women who changed employer, both before and after the first employer change, without controlling for differences in other variables. Figure 5 shows that immediately before the first employer change the average monthly incomes for men and women were 2992 and 2402 euros respectively, whilst after the first employer change the corresponding figures were 4314 and 3222 euros. Thus men achieved an average monthly salary increase of 1323 euros or 44.2% as a result of changing employer, whereas women achieved an average increase of 821 euros or 34.2%. The monthly pay advantage for men thus amounts to 590 euros (or 19.7%) before the employer change and 1092 euros (or 25.3%) afterwards.^4^

The differences are less pronounced when considering hourly wages. Figure 6 shows average hourly pay increased from 17.97 to 25.20 euros (7.23 or 40.2%) for men and from 15.20 to 19.92 euros (4.72 euros or 31%) for women, constituting hourly wage advantages for men of 2.77 euros (15.4%) and 5.28 euros (21%) before and after the first employer change, respectively. The data appear to support the second hypothesis, which predicts greater wage increases for men because they tend to be employed in labor markets which offer better prospects, regardless of how income is measured. Both monthly income and hourly wages, and absolute and percentage changes in income indicate that men, on average, benefit more from job mobility than women, at least in financial terms.

The multivariate analysis uses fixed effects models to investigate the relationship between employer change, income, and gender. Table 6 shows the regression results for two sets of two models. Models 1a and 2a contain only a variable indicating the number of employers the respondents have had so far, and an interaction between this variable and gender. The other models (1b and 2b) also include work experience and the employment characteristics already used in the logistic regression on employer change (the personal and study characteristics used there are constant over time and therefore excluded from this regression). In models 1a and 1b, the dependent variable is the logarithm of gross hourly wages, for the second set of models it is just the gross hourly wage. Being unemployed between two jobs can also affect wages (Schmelzer 2012), but is only of minor importance in this sample, which consisted mainly of young, highly educated workers in a region with low unemployment rate, especially among people with tertiary education. Furthermore, different types of unemployment cannot be distinguished with the existing data so the issue of unemployment is not addressed by the following models.

**Table 6:** Fixed-effects-regression models on hourly (log-transformed) wage. *Source:* BAP 2005/06, author’s calculations

(log.) hourly wage	Model 1a	Model 1b	Model 2a	Model 2b
1st Employer	(Reference)	(Reference)	(Reference)	(Reference)
2nd Employer	0.315***	0.227***	5.563***	4.018***
3rd Employer	0.524***	0.360***	9.328***	6.341***
4th Employer	0.738***	0.530***	12.364***	8.545***
5th Employer	0.604	0.412	10.086*	6.809
1st Employer*male	(Reference)	(Reference)	(Reference)	(Reference)
2nd Employer*male	0.031	0.011	2.145***	1.579**
3rd Employer*male	0.064	0.047	4.520***	3.949***
4th Employer*male	0.064	0.038	4.917**	4.160*
5th Employer*male	0.260	0.169	8.831	6.158
Occupational sector				
1: BIC		− 0.089*		− 3.233***
2: Manufacturing		(Reference)		(Reference)
3: Services		− 0.075**		− 1.792***
4: Media et al.^a^		− 0.140***		− 3.029***
Years of experience		0.011***		0.193***
Years of experience^a^ *100		0.021		0.031
Firm size (employees)				
Small (< 100)		− 0.043		− 0.585
Medium (100–499)		(Reference)		(Reference)
Large (≥ 500)		0.029		1.102*
Executive position		0.161***		3.600***
Public sector		0.050*		0.209
Permanent contract		0.129***		1.249**
Multinat. company		0.056*		1.007**
Constant	2.834***	2.685***	17.987***	16.217***

*BIC* banks, insurances, consulting

*N*: 146,817; * *p* ≤ 0.05, ** *p* ≤ 0.01, *** *p* ≤ 0. 0 01

^a^Media et al.: Media, education, associations; Models 1a and 1b use log-transformed gross hourly wage as the dependent variable, models 2a and 2b use untransformed gross hourly wage

The coefficients in the first column show highly significant, large, positive values for the employer spell variable, indicating large income increases after an employer change. However, there appears to be a saturation effect, as the coefficient for the fifth employer spell is smaller than that for the fourth and is not statistically significant. Further regressions were carried out using other reference categories, but the results are not reported here. These showed significant differences between all the other employment spell categories with the exception of income in fifth job, which was not significantly different from any other category. A possible explanation for this is the low number of cases: Table 4 shows that less than 1% of the participants reported having five different employers over the observation period. However other studies have also found that a large number of job changes is disadvantageous (Fuller 2008: 177), one of the possible explanation cited is that too many changes “might signal to the employers that the employees are prone to leaving their job” (Schmelzer 2012: 93).

When looking at the interaction effects, however, one can see that no significant coefficients are present there and this does not change when control variables are included. The main effect of these additional independent variables is to reduce the size of the employer spell and interaction coefficients without affecting their significance.

As noted in Sect. 5, however, one has to be careful when assessing income changes because they can be expressed in absolute and relative terms and this can affect the interpretation. For example, when there is a baseline difference in income, as here, equal percentage changes do not mean that the (absolute) pay difference stays the same; in fact under these circumstances equal percentage increases would increase the difference in wages, just as equal absolute increases in wages would reduce the gender pay gap (which is calculated as a relative difference). Table 1 shows that even in the early career there is a widening of the absolute income difference and at the same time a narrowing of the relative one. Because the logarithm of hourly wage is used as the dependent variable in the first two regression models estimated above (1a and 1b), the coefficients can be interpreted approximately as relative changes in income. That none of the interaction effects is significant therefore indicates that employer changes have no effect on the gender pay gap. This is consistent with the fact that the interaction coefficients are statistically significant when using raw hourly wage as dependent variable,^5^ as in the additional two regression models. This suggests that although changing employer delivers a larger absolute increase in income for men than women, the percentage difference between men’s and women’s incomes remains the same because of difference between their starting salaries.

The advancement hypothesis is thus confirmed with regard to absolute wage increases, meaning that men benefit more from job mobility than women. These larger gains do not, however, contribute to a widening of the gender pay gap, because in terms of relative income changes, there seems to be no significant difference between genders. Although changes of employer play an important role in income increases, the main source of income inequality can thus be traced to the differences in income which are already present in graduates’ first jobs.

## Conclusion

This article has examined two relationships: first, the relationship between gender and the frequency of employer changes, and second, the relationship between gender and the financial return on employer changes. Previous research suggested that men benefit more from job mobility but change employer at a similar frequency to women. These results were only partly replicated with the German data used in this study.

A sample of Bavarian university graduates was used to determine the frequency and effects of employer changes. The results show that women change employer more often than men, which may be largely due to the less favorable terms of their first jobs. Compared with men, women in their first job are less likely to be on a permanent contract, to hold an executive position and to work in a large company; they are also less satisfied with their first job. After controlling for variance in these and other factors, the algebraic sign of the gender coefficient in the regression model changed, indicating that men are more mobile when these variables are taken into account. Gender differences in preferences are one possible reason for this difference in mobility: If, as previous studies suggest, men are more career-oriented and prioritize income over job security one would expect them to display higher mobility because changing employer is one route to a career advancement and higher pay. This assumption could not, however, be tested with this sample, because information about individuals’ labor market preferences was not available. Additional research using different datasets is needed to address this question.

The results show that changing employer delivers large income increases for both men and women, as long as there are not too many changes over a short period. The financial return on employer change is not clearly related to gender, as only the absolute, not the relative increase in income was larger for men, thus indicating that employer changes do not affect the gender pay gap.

In summary, the job mobility of university graduates during their early career appears to have a substantial impact on their income. The gender pay gap is present at the beginning of individuals’ careers and does not increase substantially in the following years; women could potentially reduce it by changing employer more often.

Several aspects have to be considered in the context of these results. First, the sample consists of a selective group of Bavarian university graduates which is not representative of this age cohort of the German population as a whole, mainly because of the exclusion of people without tertiary education. Selectivity thus extends to important characteristics like age, education, experience, and place of residence. This means that further research is necessary to investigate the frequencies and effects of employer changes in other populations not considered here.

Second, there are different kinds of job mobility, and also changes of employer can occur in different ways. Previous works have shown the importance of rationale and volition with respect to employer changes. Involuntary changes of job seem to decrease income whereas changing job voluntarily appears to increase income (Keith and McWilliams 1995: 133). According to some authors this is only or especially the case when there is no intervening episode of unemployment (Keith and McWilliams 1999: 473; Schmelzer 2012: 93), but others have reported that indirect job mobility (i.e. when there is an intervening episode of unemployment) has a stronger beneficial effect on income (Antel 1991: 305).

Economic and family factors appear to play an important role in voluntary employer changes, with economically motivated and family-related changes having positive and negative effects on income respectively (Fuller 2008: 177). Incomes can also be positively affected by the use of firm-internal labor markets (Felmlee 1982: 149; Pavlopoulos et al. 2014: 314 f.), but gender-specific effects have been found in several of these cases. Family-related employer changes only reduce women’s incomes (Keith and McWilliams 1995: 133 f.) and women are also more likely to change employer for family-related reasons (Keith and McWilliams 1997: 331). Analysis of an Australian sample showed that for women changing employer was less likely to result in promotion and produced a smaller financial return, sometimes even a negative financial return (Johnston and Lee 2012: 149).

These findings are somewhat contradictory, suggesting that there may be national differences in labor market mechanisms. In this sample positive income effects were found although there was no differentiation between different types of employer changes (e.g. voluntary and involuntary changes) which can work in different directions. This can, perhaps, be attributed to the characteristics of the German labor market which is more strongly segmented than, for example, the British one. One of the consequences of this is that there is less downward mobility, which often decreases wages, in Germany (Scherer 2004: 373). Furthermore, losing one’s job at short notice is less common in Germany, and particularly amongst the highly educated, young respondents who made up the sample for this study; the frequency of family-related job chances is also likely to be low in this population. These factors make it far more likely that employer changes are voluntary and hence yield an increase in salary.

The finding that the gender pay gap does not diminish over time may also be due to the specific characteristics of the German labor market, in which field of study has a strong impact on entry into the labor market and entry job characteristics have long-lasting consequences (Scherer 2004: 378). It is therefore plausible that the gender pay gap is present at graduation—largely because of gender differences in self-selection of field of study and hence occupational sector—and does not diminish in subsequent years. Nonetheless, additional analyses based on more extensive samples including individuals with fewer academic qualifications, older individuals, and data on the reasons for employer change, should be conducted in order to identify the effects of labor market mobility.

## Footnotes

1 **D**eutsches **Z**entrum für **H**ochschul- und **W**issenschaftsforschung—German Centre for Higher Education Research and Science Studies.

2 Participants were asked to regard a job change as a new spell of employment if it involved a change of employer or another important characteristic. It is likely that not everyone reported all relevant changes, so the incomes of immobile respondents are probably underestimated. To take account of this all analyses were conducted twice: the second time, the income of participants who reported one spell of employment and no change in income was increased by 2% every 12 months. The only effect this had on the results was to reduce the income advantage for mobile employees relative to immobile employees.

3 To test these findings, alternative model specifications (exponential model, Weibull model) were tested; they yielded similar results.

4 Incomes appear to decrease prior to the employer change; this is caused by self-selection. Better working conditions and higher pay act as a disincentive to change job (see Sect. 6.1), hence people on lower salaries make their first change in employer at an earlier stage. This means that there are fewer observations of this population at higher negative numbers (e.g. if an individual changes job after 10 months there will only be 10 months of observations prior to the change, if an individual does not change jobs until he or she has been employed for 20 months there will 10 additional months of pre-change observations). Hence people on higher incomes are over-represented at the early points in time, leading to higher average incomes and lower numbers of observations at employer change minus 20 months than at employer change minus 1 month.

5 Using raw gross hourly wage is, of course, potentially problematic because of the usual linear regression assumptions so these results should be considered with care. However, not least because they are consistent with the other results presented in Sects. 5 and 6.2, they provide certain evidence.
